# How to establish and maintain a multimodal animal research dataset using DataLad

**DOI:** 10.1038/s41597-023-02242-8

**Published:** 2023-06-05

**Authors:** Aref Kalantari, Michał Szczepanik, Stephan Heunis, Christian Mönch, Michael Hanke, Thomas Wachtler, Markus Aswendt

**Affiliations:** 1 University of Cologne, Faculty of Medicine and University Hospital Cologne, Department of Neurology, Cologne, Germany; 2 Psychoinformatics Lab, Institute of Neuroscience and Medicine, Brain & Behaviour (INM-7), Research Centre Jülich, Jülich, Germany; 3 Institute of Systems Neuroscience, Medical Faculty, Heinrich Heine University, Düsseldorf, Germany; 4 Faculty of Biology, Ludwig-Maximilians-Universität München, Planegg-Martinsried, München, Germany; 5 Cognitive Neuroscience, Institute of Neuroscience and Medicine (INM-3), Research Centre Jülich, Jülich, Germany

**Keywords:** Research management, Translational research, Research data

## Abstract

Sharing of data, processing tools, and workflows require open data hosting services and management tools. Despite FAIR guidelines and the increasing demand from funding agencies and publishers, only a few animal studies share all experimental data and processing tools. We present a step-by-step protocol to perform version control and remote collaboration for large multimodal datasets. A data management plan was introduced to ensure data security in addition to a homogeneous file and folder structure. Changes to the data were automatically tracked using DataLad and all data was shared on the research data platform GIN. This simple and cost-effective workflow facilitates the adoption of FAIR data logistics and processing workflows by making the raw and processed data available and providing the technical infrastructure to independently reproduce the data processing steps. It enables the community to collect heterogeneously acquired and stored datasets not limited to a specific category of data and serves as a technical infrastructure blueprint with rich potential to improve data handling at other sites and extend to other research areas.

## Introduction

Data management and sharing require best practices as recently introduced for human MRI^1,2^. In our experience, most laboratories rely on non-standardized data storage on local hard drives or network drives with insufficient user management and backup capacity. Despite the fact that only a minority of MRI studies are using small animals, it is alarming that on OpenNeuro, a widely used neuroimaging data-sharing platform^3^, only 3% of datasets contain data from mice or rats. Similarly, on another popular data-sharing platform, not specific for neuroimaging, Zenodo^4^, only about 30% of MRI datasets are from mice or rats. In addition, it is surprising and contrary to FAIR principles^5^, if in the majority of these neuroimaging datasets, only the imaging data are provided. This excludes a large part of the accompanying data, e.g., the microscopy files used for *in vivo* cross-validation. We also identified a clear lack of step-by-step guides or automated routines required to reproduce the processed data. These examples underline previous reports^6^ that small animal data sharing is far from common and that there is no standardization in terms of data acquisition, storage, and sharing. If data are not shared and thus not available for re-use as it is the case for 93% of biomedical open-access publications^7^, this also contrasts strongly with the 3 R principle of minimizing the number of animal experiments^8^. Therefore, it remains very difficult to compare studies between different laboratories, which contributes to the reproducibility crisis^9^, and small animal (neuroimaging) studies are no exception^10^.

We envision a change toward the conditions of good scientific practice and the principles of FAIR - Findable, Accessible, Interoperable, Reusable^5^ and Open Science^2^ to improve the reliability and recognition of animal studies. Our goal was to create an easily applicable approach for setting up a multimodal dataset that provides access to raw and processed data, methods, results, and their provenance. Proper research data management (RDM), as it is also increasingly required by funding agencies and publishers, is key to meeting these standards^2,11,12^.

Here, we describe our strategy for data organization, metadata collection, and data/analysis tracking using three established tools: our relational database^13^, the data platform GIN (G-Node Infrastructure services, https://gin.g-node.org), and the research data management software DataLad^14^. The database is used to collect all experimental metadata about the complete timeline of longitudinal and multimodal animal experiments, including MRI, histology, electrophysiology, and behavior. GIN and DataLad are both based on Git, a popular version control system, and git-annex, which extends Git’s capabilities, especially with respect to managing large files. GIN is an open-source, web-based data management service with various features for collaborative data handling, e.g., built-in versioning, secure access, persistent data identifiers for publication (DOI), automatic indexing, and data validation. DataLad is a data management software designed to support the various stages of the development of digital objects. Importantly, DataLad can be seen as an overlay on top of existing data structures and services: Tracking files does not change the files themselves or the location from which they can be retrieved by data processing tools.

## Results

### Workflow

**Fig. 1:**
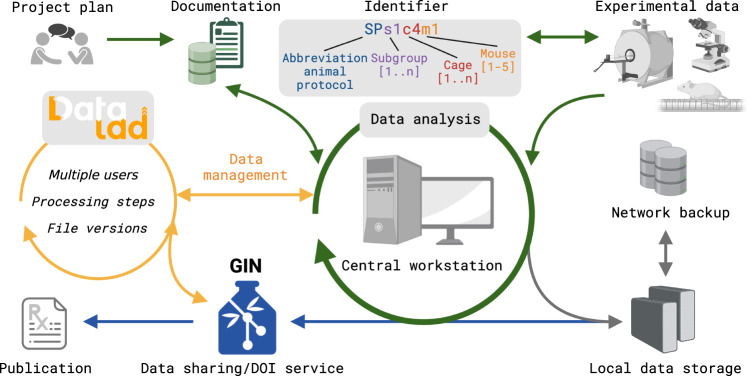
Green arrows: workflow for project planning, data acquisition, processing, storage; gray arrows: backup plan on local and network storages; orange arrows: integration of DataLad for version control; blue arrows: publication process using GIN as the online hosting service. Figure created with Biorender.com.

**Fig. 2:**
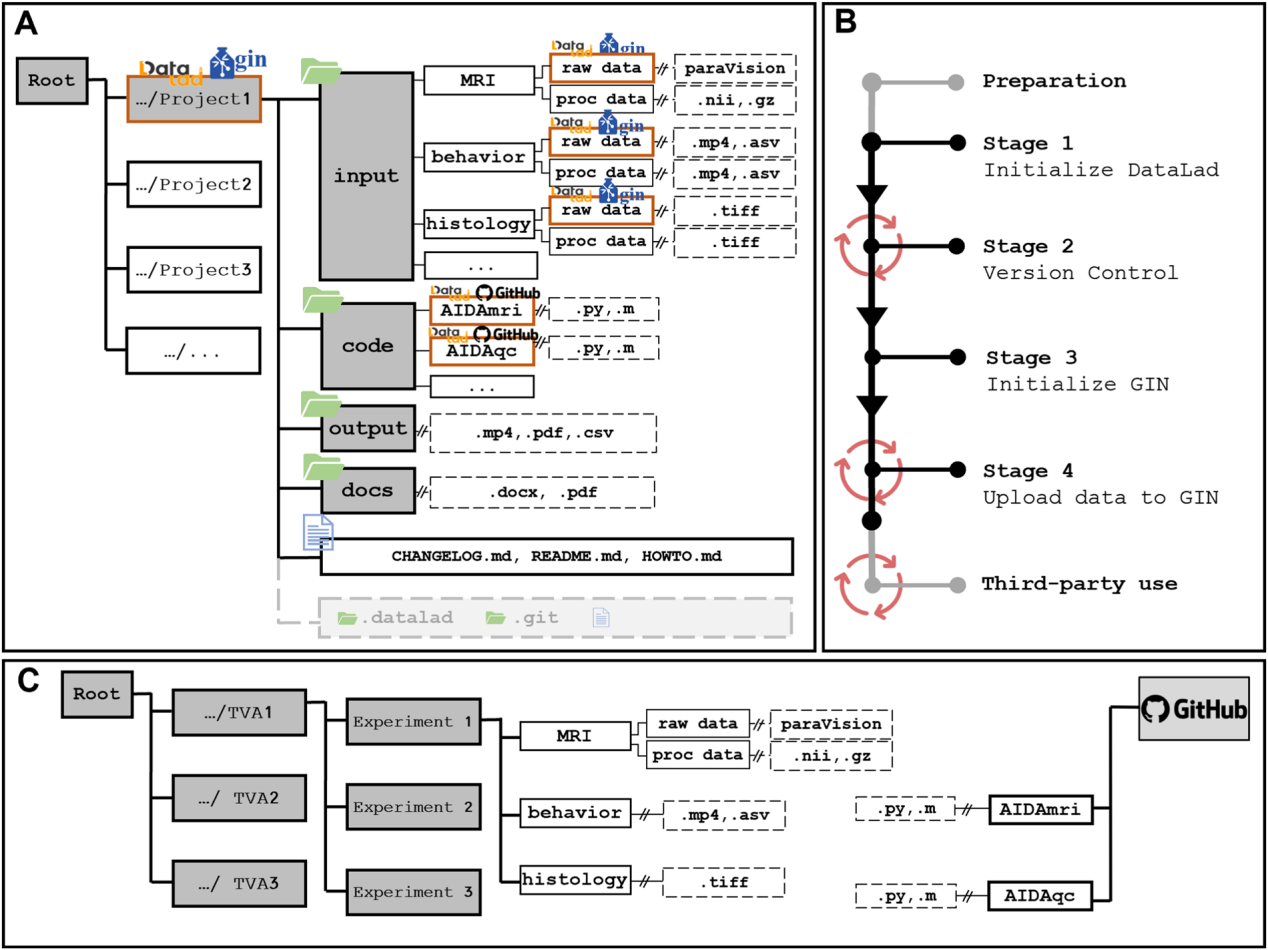
(**A**) YODA-directory structure and integration of DataLad. The root directory of the operating system (OS), the list of projects, and project-related content (folders + file types). The dashed and “//” separated connections indicate that there can be additional levels, e.g., the time of measurements. During the conversion of the Project1 folder into a DataLad dataset, the corresponding DataLad files, (gray boxes), are created as additional information in the folder, without affecting the rest of the data. “raw_data” folders and all folders in “code” are independent subdatasets in the context of nesting and establishing decentralization with “code” being located in Github instead of GIN. (**B**) Step-by-step guide for creating the DataLad dataset is composed of 4 mandatory subsequent stages and framed by an initial preparation phase and an optional third-party use scenario (red circles highlight recurring stages). (**C**) Folder structure based on the permit of performing animal experiments without DataLad (TVA = Tierversuchsantrag (German: animal protocol). This is the old structure before the YODA structure was implemented, all processing was also done within this structure, using the pipelines parallelly from another storage separated from this structure. The original raw data is still kept in this structure as an archive of raw data, but the rest is abolished.

Over the last 5 years, we have established a strategy for 1) project planning, 2) documentation, 3) data acquisition and storage, and 4) data sharing (Fig. 1). Project planning and experimental details are recorded in an in-house relational cloud-based database^13^. A key element for both the database and the data storage is the identifier, the study ID for each animal, used in a standardized file name structure to make the data findable. The directory structure for the raw data follows the permit of performing animal experiments. The data for a specific project is organized following the YODA principles (https://handbook.datalad.org/en/latest/basics/101-127-yoda.html), which is compatible with existing standards, e.g., the BIDS structure^15^ (Fig. 2A). An automatic incremental backup routine was installed, which transfers the data from an external diskstation linked to the main analysis workstation to a centrally managed network drive. In preparation for publication and to facilitate data reproducibility, the experimental raw and processed data is made publicly available on GIN, and post-processing details and pipelines are specified - either in the publication or on a GitHub page (https://github.com/aswendtlab/Project_C3a_peri-infarct).

DataLad is used as the central data management tool (Fig. 1) and for version control: It keeps track of which files were modified, when, and by whom, and provides the ability to restore previous states. To this end, DataLad is agnostic of the data type and provides a unified interface for managing code and data files (typically handled by Git and git-annex, respectively).

### Dataset nesting

Within DataLad datasets, it is possible to nest (an unlimited number of) other DataLad datasets, each sub-dataset remaining a standalone component with its own history and siblings. To achieve this structure the following stages 1 through 4 can be performed independently within an established dataset. In this way, the user can subdivide the project e.g. by publication, data type, or storage location. To clarify this, it is possible to place a top-level dataset together with the sub-datasets in the online repository service. Here, the data tracked by a dataset directly is stored as usual, but the sub-datasets are available only as links to their own repository, which can, but do not have to be hosted by the same online repository provider. In our case, all of the project code is stored and maintained in GitHub repositories (https://github.com), and installed as multiple sub-datasets within the main top-level dataset **Project1** on GIN (Fig. 2A). The structure introduced by the Yoda principles makes the entire project self-contained. No further element outside the project is needed to get from inputs to results. In contrast, no self-containment is possible for the structure in Fig. 2C.

INFO: If a dataset or a subdataset is to be implemented for a folder that contains only coding scripts, stages 1 to 4 should be slightly optimized. Briefly, git is much better suited for version control of scripts that contain code. When step 1.2 is run, everything is handled by git-annex by default, the alternative is to use datalad create --force -c text2git. The additional configuration puts git in charge of the contents of the datasets.

### Step-by-step data management protocol

Based on our workflow development (Fig. 1), this use case description provides a step-by-step guide for *non-expert users* on how to manage large experimental datasets containing complex, multimodal files, i.e. the raw and processed data as well as the code and output files.

For the hard- and software requirements and installation routines, see the Methods section and Supplementary Material. The workflow consists of 4 mandatory stages (Fig. 2B): 1. Initialize a DataLad dataset, 2. Version controlling, 3. Initializing the remote (online) repository, and 4. Uploading to the remote repository. We also describe an optional use-case scenario for third-party use, i.e., collaborating on the same dataset with other researchers, and dataset publication.

#### Preparation

In order to create the data structure, start with the main **Project** folder and sort the raw data in the related **input** folder (Fig. 2A).

When converting the **Project** folder to a DataLad dataset, the corresponding DataLad files are created automatically without changing the files and structure. DataLad files contain the version and the history of all files in the project folder. **Project1** serves as a super-dataset with the associated **raw_data** and **proc_data** folders as sub-datasets with their own repositories on the repository services GIN and GitHub, respectively. With this structure, DataLad can register actions such as processing **raw_data** from a specific pipeline in **code** and store the output of the pipeline in **proc_data**. These actions are saved in the history information of the super-dataset **Project1**.

In our case, the data from multiple animal protocol folders are copied to the project folder and the original raw data (in the archive) remains untouched. This process can be tracked if Datalad is already effective for all storages. In case of other data structures, e.g. using PACS server, the transfer of the data into the main **Project** folder would be similar. The idea is to have all project-relevant data available for analysis and later on sharing. Each sub-folder of **input** is named according to the methodology (e.g., MRI, behavior, histology, etc.) and can contain various file types (Table 2). The other folders include the **code, results**, and **docs**. In this example, **code** contains the Atlas-based Imaging Data Analysis tools AIDAqc and AIDAmri^16^ for automated quality control, processing of multimodal MRI^16^, and registration with the Allen Mouse Brain Atlas^17^. The **docs** folder contains all necessary (metadata) information and documentation to reproduce the MRI dataset (e.g. which study ID belongs to which experimental group, time points, MRI scan type, etc.).

The following stages are executed to start the conversion of the **Project1** folder into a DataLad dataset (Fig. 2B). A video guide is available (https://gin.g-node.org/Aswendt_Lab/Example_Dataset_Kalantari_Paper/src/master/doc).

#### Stage 1: Initialize a datalad dataset

1.1 To begin, open the terminal and change the directory to the target folder. In this example, it is **Project1**.


»cd Project1


1.2 Once in the target folder, run the following command to create the DataLad dataset-specific folders and files in the **Project1** folder (Fig. 2A).


» datalad create–force


**INFO:** DataLad provides numerous **commands** and each command has different **options**. You can create an empty DataLad *dataset* folder by datalad create folder_name. Here, in step 2, the create command was used to initialize a DataLad *dataset* in the current working directory, and the --force option allowed dataset creation in a folder already containing data. For more information about other options you can use--help.

#### Stage 2: Version control (local data)

2.1 After the initialization stage 1, continue with the following step. Note that this step may take some time depending on the hardware^1^.

(^1^Refers to features such as internally or externally connected storage drives, network drives, storage types such as hard disk drive (HDD) or solid state drives (SSD)).


» datalad status»datalad save -m "user message"


Once the dataset is created, steps that modify its content are recorded by executing the datalad save command. Stage 2 shows the first practical use of DataLad datasets: recording changes. In our case, the **raw_data** folder contains, e.g. MRI raw data. After processing using AIDAmri^16^, the results are stored in the **proc_data** folder (Fig. 2A).

**INFO:** The datalad status command can be considered as an inspection tool that prints the current status of each file, whether it is tracked or untracked by DataLad, deleted or modified. After initializing in step 2.1, all status printouts would show “untracked”. Any newly added files will not be tracked until datalad save is explicitly executed. After a file has been saved and the contents subsequently changed, the status will be printed differently. In general, datalad status is used for informational purposes only and may be helpful in identifying recent (unsaved) changes.

The datalad save command, on the other hand, records new changes in the.git folder. The save command also has several options, where -m associates the save with a user message which can describe the purpose of the change. These messages can be beneficial for the user to find a particular version or to recall changes in the dataset more easily. If you are interested in more options for a command, use the --help option. For example, datalad save --help displays all available options for the command save.

2.2 (Optional) If changes are done programmatically (e.g. by running a custom script), this can be done with a command like the following:


» datalad run -m “user message” -–input … python < code.py >


The run command registers information about the input and output data of the executed code (here a Python script) and registers it in the history information of the dataset. The datalad save command is automatically executed when using datalad run, but in addition to the user-provided message, a re-executable run record is also stored to capture provenance.

It is important to emphasize that Stage 2 is a repetitive Stage (Fig. 2B), i.e., it can be repeated to record consecutive changes or new states. Over time, this process creates a logbook, in which all the actions that a user has performed on the project over time are recorded. How to access and use this logbook is explained in the following step.

2.3 All registered information about the recorded changes can be accessed by:


» git log


This will display all the changes that have been made since version control began. Optionally by typing:


» git log -2


**Fig. 3:**
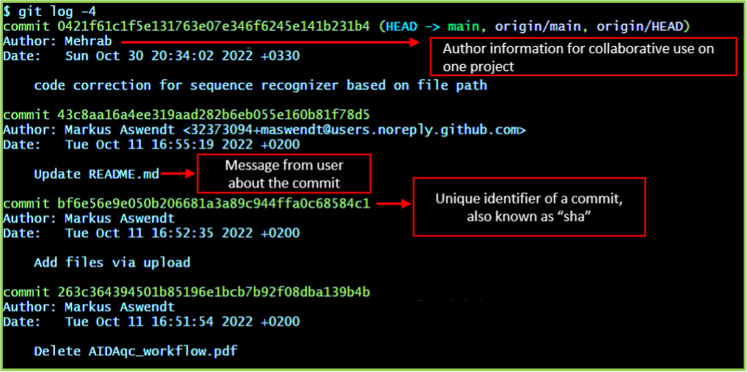
Sample git log output of a dataset showing information about the author, date, changes, and the corresponding commit identifier, also called “sha”.

Only the last two changes or **commits** will be printed (Fig. 3).

**INFO:** A **commit** is a snapshot of the current state of the project. The difference between two commits in one datalad dataset means the changes that have happened between those two states of the project. Some data might be produced, changed, moved, or deleted. There are other ways to access the history such as **tig** (https://jonas.github.io/tig/) or some with their own graphical user interface (GUI) like **gitgui,** **gitk**, etc (https://git-scm.com/downloads/guis).

#### Stage 3: Initialize a GIN repository as a DataLad dataset sibling

In this scenario, we are using the data platform GIN (other options are available, see Table 1). A prerequisite for the following steps is an operational GIN account (https://gin.g-node.org/user/sign_up) with an associated ssh key pair (http://handbook.datalad.org/en/latest/basics/101-139-gin.html) between the GIN account and the account on the local computer. Note: This step may take some time depending on the network speed.

3.1. Create a repository with a project-specific dataset name under the GIN account and copy the project-specific ssh link generated by GIN.
3.2. Execute the following command with your own credentials in the Project1 folder.

**Table 1:** Overview of currently available research data platforms, data hosting services, and cloud storage serving different needs for data storage, version-control, metadata collection, and or sharing.

Type	Name	Storage space limit/ File size limit	Open source/ Costs	Data types	Versio-ning	Meta- data	DOI Service	Reference/Link
Research data platform	GIN	No/No	Yes/No	No restrictions	Yes	Yes	Yes	https://gin.g-node.org
Research data platform	EBRAINS	No/No	No/No	No restrictions	Yes	Yes	Yes	https://ebrains.eu/service/share-data/
Data hosting	Harvard Dataverse	1TB/2.5GB	No/No	No restrictions	Yes	Yes	Yes	https://dataverse.harvard.edu
Research data platform	OpenNeuro	No/No	No/No	MRI, PET, MEG, EEG (BIDS format only)	Yes	Yes	Yes	https://openneuro.org
Research data platform	OSF	50GB/No	Yes/No	No restrictions	Yes	Yes	Yes	https://osf.io/
Data hosting	Zenodo	No/50GB	No/No	No restriction	Yes	Yes	Yes	https://www.zenodo.org/
Data hosting	Dryad	No/No	No/No	No restrictions	Yes	Yes	Yes	https://datadryad.org/
Data hosting	figshare	1TB/20GB	No/Yes	No restrictions	Yes	Yes	Yes	https://figshare.com
Cloud storage	Amazon S3	No/5TB	No/Yes	No restrictions	Yes	No	No	https://aws.amazon.com/
Cloud storage	Google Drive	20TB/5TB	No/Yes	No restrictions	No	No	No	https://www.google.com/intl/eng/drive/


datalad siblings add–dataset.–name gin–url git@gin.g-node.org:/username/dataset-name.git


**INFO:** datalad siblings is used for all actions that affect other copies of the dataset. Here the add option is used to link the dataset to a new location where the siblings or the copy of the dataset should go. Next, --dataset defines the path of the dataset being configured, here it is defined by the “.”, meaning the current folder location. --name is the name of the sibling which can be defined by the user. However, it is logical to name it according to the online repository service for ease of use and to avoid confusion. Here we use gin. The --url option is for the link available on the project page acquired in step 3.1, which is generated explicitly for the project created there. For some repositories, like GIN, dedicated commands are provided that automate the creation and registration of remote siblings in a single step. For GIN this command is create-sibling-gin.

#### Stage 4: Upload data to GIN

After the remote repository is set up and a connection between the two is established, the project can be uploaded (pushed) to GIN.

4.1. Start by entering the following command in the terminal:


datalad push --to gin


Just like stage 2, this step can be repetitive (Fig. 2B). As a dataset and its history information progress, new commits are added, and updates can be pushed to the remote repository. This is done efficiently, meaning that only changed files are transferred. Versioning makes the state of the uploaded dataset unique. In addition, GIN is fully compatible with git and git-annex, and its web interface can, for example, display the history of changes and associated commit messages.

Depending on the size of the project and the quality of the Internet connection, the execution of this step may take some time. Therefore, it may be wise not to try to push all the data in one batch, i.e., split it up and do the push process in multiple batches. This is possible by adding the path of a specified smaller part of the project to the command:

4.2 (Optional) datalad push --to gin <path_to_smaller_project_part>

### Working with published data

Anyone interested in the project can now access and download not only the data but also the history of the project as it has evolved over time, through what is called “cloning”. From a third-party perspective, the first step is to visit the respective repository on the GIN website, where the SSH link or the HTTPS link can be found respectively, depending on whether the dataset is to be modified or only downloaded.

**INFO:** On the projects page of a GIN account, there are three links for accessing the repository, SSH, HTTPS, and GIN. In simple terms, SSH and HTTPS are different ways of communicating between the user’s operating system and the online repository service. The main difference in our use case is that an SSH connection is required if we want to upload or “push” the data to the remote repository. For downloading or “cloning” the data, an HTTPS connection is sufficient. As briefly mentioned in stage 3, setting up an SSH link requires additional steps, but HTTPS links can be used without further concern.

1. Copy the SSH/HTTPS link from the GIN project web page.
2. Open the terminal and navigate to a folder where the project content should be located and run the following command, replacing the example URL with the copied link.
  For SSH links:
  datalad clone git@gin.g-node.org :/username/dataset-name.git
  For HTTPS links:
  datalad clone https://gin.g-node.org/username/dataset-name
  If the HTTPS link is chosen, note that the link does not have a.git extension.
  **INFO:** A useful feature of the datalad clone command is that it does not download the entire dataset at once. It downloads only the folder structure, the small files, and the filenames of large files, i.e., files that are handled by git-annex. The datalad get command can then be used to selectively download the contents of large files so that the entire content becomes available locally. This can be useful from two points of view: first, if the entire dataset is very large and only some parts are of interest, only those parts can be selectively downloaded. Second, if only the project structure and file name are of interest, in this case, datalad get would not be called at all.
  If you want to download the entire project, continue with the following command in the DataLad dataset folder created after the last step.
3. Type in the following:

datalad get


The command can be restricted to a specific path (directory or file) to selectively retrieve the contents.

As introduced in the previous section, dataset nesting can be very useful from a third-party perspective. In our example, the top-level dataset **Project1** contains multiple datasets for the “raw_data” folders and different code pipelines (Fig. 2A). The intention for this setup was, first, to make the raw data and codes separately available to third-parties without having to download all the peripheral project structures, and secondly, to preserve updatability of the various pipelines in the **code** folder, i.e., as the pipelines are updated over time, cloning the top-level dataset automatically associates the most up-to-date pipelines with it.

### Metadata collection and dataset publication

Since our dataset is intended for publication and reuse, it is critical to annotate it with relevant information. On creation, GIN repositories are by default private, i.e., access restricted, but can be set to public through a tick box in the settings menu. Public datasets on GIN can receive a unique persistent Digital Object Identifier (DOI), which makes them citable. Next to the publication metadata, we include documentation about the dataset, such as experimental groups and time points, as well as modality-specific information, e.g., about MRI sequences, which is retrieved from our relational database.

## Discussion

Here, we provide a step-by-step guide for non-expert users to implement a FAIR data workflow applicable to small animal data. In this workflow, we are using a simple but efficient local backup scheme and standardized data structure in combination with GIN as a solution for public data availability. DataLad was utilized as a data management tool to provide a foundation for transparent and reliable collaboration between researchers, with the intention of encapsulating all elements of a project, such as input data, codes, and results, along with information about how they are connected^18^.

Our motivation to implement this workflow was to ensure data preservation, efficient collaboration, data sharing, and increased reproducibility. Such practical needs related to research data management are widespread in the field and so far only single solutions have been proposed (Kuhn Cuellar *et al*.^19^). A recent survey on data management and sharing in (human) neuroimaging shows the significant challenges associated with properly managing and sharing neuroimaging data, with the biggest limitations being time and lack of best practices^20^. Researchers consider themselves less mature in data sharing than in data analysis and collection practices. To overcome this limitation, our focus was on an easy-to-implement workflow that uses only freely available software and does not require any special prior knowledge.

A standardized subject identifier and file/folder structure (see Methods section) are the foundation of efficient research data management. The created identifier for each animal and standardized file naming helps to make the data traceable even in the absence of other metadata. In our case, the animal protocol determines which and how often a method can be applied. In order to provide full transparency according to the guidelines of good scientific practice using animals^21^ and to simplify documentation for the audits by the local authorities, we collect all experimental data in an electronic relational database^13^ and store the raw data in the structure predefined by the animal protocol. This raw data directory serves later on as an archive, which remains untouched. For the project/publication-related processing, all raw data - which can come from different animal licenses - is copied into the input folder to leave the raw data untouched for security while providing maximum flexibility to work with the data. The YODA structure contains not only the **input folder** but also **output,** **docs**, and **code**. Here, our approach differs from previously suggested folder structures with a 3-level hierarchy, i.e., laboratory level (organization holding several projects), projects level, and subsequent experimental level^22^. For publication or collaboration, the whole YODA structure is uploaded to the online data hosting service, in our case GIN. Importantly, DataLad is flexible in where the data is stored. If users have their complete data in alternative storage solutions such as on XNAT or PACS server for radiological data^23^ or OMERO^24^ as for microscopy data, there are dedicated DataLad extensions (https://docs.datalad.org/), or additional extensions can be developed with minimal effort to support additional services. Our workflow is not excluding other software and data hosting services, for example, CKAN (https://ckan.org), Harvard Dataverse (https://dataverse.harvard.edu), and Barcelonaβeta Brain^25^, with their own strategy to handle complex multimodal research data. In contrast, it provides a versatile and interoperable RDM, with the necessary flexibility and simplicity to be adapted to local experimental paradigms and existing IT infrastructures in small laboratories to large multisite consortia^12,18^.

### Metadata

Metadata is a very important ingredient in current research practices, especially when it comes to transparent collaboration between researchers according to FAIR and neuroimaging community-specific guidelines^5^,^26^, For MRI saved as DICOM or NiFTI file formats, there have been several attempts to make the header file more metadata rich, with the BIDS standard being the most advanced and widely used option for structuring files according to MRI sequences and including standardized metadata^15^. Our workflow is fully compatible with the BIDS format (see example in 10.12751/g-node.3yl5qi^27^) but we decided to make BIDS no requirement to use the workflow as for example is the case for the data platform OpenNeuro, to maximize the flexibility for researchers working with different file formats. Although we fully support the efforts to maximize standardization at the level of metadata, in our own experience working with multimodal data (from MRI, behavior, electrophysiology, and microscopy), it is necessary as an intermediate step, to prioritize the existence of as much metadata as possible in machine-readable files (txt, csv, json), which do not necessarily have to be the current community-based structure. We, therefore, recommend using the workflow in combination with a relational database such as our own development^13^, REDcap^28^, or others, which allows the generation of such machine-readable files. With basic programming expertise, it will be easy for third-party users to search and filter the shared data based on these files.

If the processing requires multiple steps or processing tools, we further suggest to include a more detailed step-by-step guide (e.g. https://github.com/aswendtlab/ Project_C3a_peri-infarct), which goes beyond the requirements of metadata reporting in existing standards, i.e. BIDS. Such step-by-step guides might be necessary for data replication in case automated routines, i.e., datalad run, cannot be used.

### Limitations and Outlook

It should be mentioned that DataLad requires some upfront investment of time and resources, but the efficiency-, quality-, and reliability gains down the line make those investments worthwhile in our experience.

The inability to reproduce the results of a particular study is not unique to neuroimaging in small animals. This is mainly due to the lack of transparency and methodological details, such as a step-by-step protocol in scientific papers. Reproducing a study requires access to a variety of other documents, such as scanning parameters, pre-and post-processing procedures, and individual subject characteristics. To this end, in addition to the workflow presented here, we take a pragmatic approach, i.e., we make the raw and processed data publicly available on GIN and specify the post-processing details on a project-specific GitHub page.

Converting the data folders into a DataLad dataset not only provides the benefits described above, i.e., track modifications, easy sharing of datasets by publishing them to siblings, and efficient management of large files by reducing transport and storage needs. As stated before DataLad opens up your datasets for much broader use and provides easy reuse of published datasets in other DataLad datasets through the DataLad subdataset mechanism. DataLad also offers provenance tracking (re-executable annotation of changes), and metadata management. Together with DataLad’s run-command and rerun-command, which allow “tracked execution” of operations on a dataset, DataLad enables truly reproducible research^29^.

### Conclusion

Open data sharing embedded in a proper RDM protocol will not resolve shortcomings in study design or analysis strategy, but most importantly it provides other researchers to identify potential shortcomings and address them in future work, which prevents the repetition of errors^20^. Our efforts serve as a blueprint for other areas of preclinical imaging with a high impact on the research practice in the animal neuroimaging community and beyond. In line with the 3 R principle to reduce the number of animals, this workflow enables the community to collect heterogeneously acquired and stored datasets to foster mouse brain simulation experiments and implement a cross-species modeling approach. Moreover, the close collaboration with efforts for data and metadata standardization, provenance tracking and workflow management, integration into an interoperable research data platform ecosystem, and specification of the computational model using standardized means will help to strengthen the interaction of all participants and will ameliorate the current translational gap between basic and clinical researchers.

## Methods

In this section, we describe the details of the material necessary to reproduce the workflow.

### Hardware

The workflow described here (Fig. 1A) was developed using a Mac Pro (Late 2013) as the main workstation run in server mode. In this way, multiple users can access the Mac via the built-in screen or file sharing (VNC/SMB protocols) and retrieve data and run programs in parallel. A similar way of working is possible with workstations running Linux or Windows 10/11 installations via remote desktop connections. The data storage strategy was developed according to best practices^30^ including a backup plan, different backup locations, backup validity checks, and the possibility to extend data storage. The main local storage - directly connected to the workstation - is a 20TB LaCie Thunderbolt drive operating in Raid-5 mode (one hard drive can be damaged while the file system remains intact). All data are first manually copied to this local storage by the responsible experimenter. The path to the raw data is documented in the electronic database^13^. Automated and incremental backups are performed using Carbon Copy Cloner (Bombich Software, Inc., USA) weekly from the local storage to network storage (managed by the IT Department of the University Hospital).

### Software

The workflow (Fig. 1A) builds upon free and open-source software: Python (https://www.python.org/, RRID:SCR_008394), GIN (https://gin.g-node.org, RRID:SCR_015864), and DataLad (https://www.datalad.org, RRID:SCR_003931). Other data hosting options exist (Table 1), which can be easily integrated in the provided workflow. Representative installation guides are provided in the Supplementary Material. For the most up-to-date installation instructions, see the related websites.

#### DataLad

DataLad is used for distributed version control: creation, synchronization, and tracking of linked dataset copies (called siblings). A DataLad dataset can have one or several siblings, stored either locally (backup drives, local servers, different workstations) or online (e.g. GIN). Changes made to one dataset copy can be synchronized with other copies, and synchronization is always explicit (it is easy to know whether versions diverge or not). File content availability is tracked, and content available remotely can be retrieved on demand to save space locally. This also means that file exchange, backup, and publishing are all done through the same software interface, even if the locations are different.

#### Definitions

1. Repository**: Folder with files and subfolders as a structural unit for code or data management;** can be any set or combination of files with different folder structures and different data types, sometimes it can even be empty and contain no files.
2. **DataLad dataset:** repository on which DataLad is executed and version control is running.
3. **Initializing a DataLad dataset:** to create an empty dataset that is subject to DataLad’s version control. If files are added to this dataset after that, they will be tracked by DataLad.
4. Initializing **DataLad in an already existing repository:** the transition of a dataset in a file/folder structure to a DataLad-managed dataset.
5. **Tracking/version control:** if a file changes because it has been replaced or edited by a user, DataLad logs those changes over time accordingly.
6. **A DataLad sibling/clone:** can be defined as a copy of the dataset. This does not necessarily mean that it is an exact copy, or that all the data is fully available. A placeholder is a file with the same name as the original file but without its content.
7. **Git:** a free and open-source version control system used to handle small to very large projects efficiently
8. **Git-annex:** git-annex is a distributed file synchronization system that aims to solve the problem of sharing and synchronizing collections of large files.
9. **Dataset nesting:** datasets can contain other datasets (subdataset), which can in turn contain subdatasets, and so on. Every dataset that contains another subdataset can be called a **superdataset**. The **top-level dataset** is the superdataset which has the highest level in the hierarchy of datasets.

#### Data storage solutions

Online data hosting providers offer the infrastructurefor researchers to upload their data and share it with others. These services differ in their primary focus: cloud storage (file storage and limited sharing, no metadata indexing), simple data hosting services (long term hosting, automated metadata indexing), and research data platforms (long term hosting, sharing, collaboration and data management) (Table 1).

Services and platforms that provide persistent identifers (DOI) are key to adopting practices of FAIR and open science in line with aspects of reproducibility and data reuse. However, only a minority of studies share their data, which might change with the increasing demand from publishers and funding institutions. We have chosen the research data platform GIN (https://gin.g-node.org/G-Node/Info/wiki/), which is supported by the German government (BMBF Grant 01GQ1302) and LMU Munich, and developed open-source by the German Neuroinformatics Node (G-Node). GIN is a registered resource (10.17616/R3SX9N) and fulfills the criteria for repositories and scientific gateways endorsed by the INCF^31^.

To make a dataset eligible for the GIN-DOI service, specific metadata, including a GIN-specific file called “datacite.yml” needs to be created, which contains information about the authors, title, description, keywords, and license according to the DataCite schema (https://schema.datacite.org/) need to be provided. On GIN, this metadata needs to be in a file called “datacite.yml” in the root of the repository. A license should be chosen (e.g., https://creativecommons.org/choose/) to specify attribution, derivatives, and sharing requirements, with the license text to be included in a text file called LICENSE. The GIN DOI service ensures permanent access to the dataset using a persistent identifier (https://gin.g-node.org/G-Node/Info/wiki/DOI).

INFO: The choice of a data hosting service should be made carefully, as various factors play a role, e.g. the availability, anonymity encryption, the general target group of external or internal users, the scope of the data, frequency of usage, etc. Importantly, DataLad will work with all of the listed services. DataLad version control is applied regardless of where the data resides, i.e. locally or in an online data store. Nevertheless, an adequate backup is necessary in order not to lose access to the data, the DataLad records and the associated history. Therefore, it is common to store a replica of the dataset and its version-controlled history somewhere else with sufficient storage space.

### Data structure/data types

#### Folder structure

We are working with two data structures, one in which the (raw) data is stored according to the experiments described in the approved animal protocol (Fig. 2C), and the second one in which the project-specific data is stored (Fig. 2A). First, we collect all raw data in a uniform folder structure according to the main projects and subprojects in our animal experiment licenses and as approved by the local authorities.A typical raw data folder (MRI/raw_data/P1/SPs1c4m1_1_1_20180105_094610) specifies the experiment (MRI), the type of data (raw data), the time point (P1, i.e., post-stroke day 1), the studyID (SPs1c4m1), and a MRI hardware (Bruker) specific code (i.e., study number, reconstruction number, date as YYYY-MM-DD, and time as hh-mm-ss). We recommend saving the raw data in the folder structure related to the animal protocol, which simplifies documentation for the authorities. In a second step, before initializing DataLad, the raw data is copied from one or multiple raw data folders in the YODA structure for each project/publication. The YODA structure ensembles data input, output, docs, and code in a project-specific structure (Fig. 2A).

#### Identifier

In order to keep track of the large number of experiments, we created unique identifiers for each mouse/scan (Fig. 1). The identifier (Study ID) combines elements of the animal protocol, the (sub)project, the cage, and the animal number per cage. For example, **SPs1c4m1** relates to project SPasticity, subproject 1, cage 4, mouse **1**. In principle, other information, such as sex and genotype can be added according to common nomenclature, e.g., **SPs1c4m1mRbp4**, relating to a male (m) Tg(Rbp4-cre)KL100Gsat (short Rbp4) mouse. Importantly for translational studies, the study IDs should not be modified to reveal the experimental group to the user, i.e., the user remains blinded during data collection and analysis. In any case, the main functions of the identifier should be preserved, i.e., to anonymize the subject (in terms of experimental details) and to remain identifiable by keyword-style search/sort terms.

**INFO:** The IDs refer to the animal protocol and are stored in a fixed folder structure similar to that used in the BIDS format. Our file convention for naming and structuring files was specified before BIDS became popular for animal MRI data. Therefore, we have datasets with underscores and hyphens as separators, e.g. TP_T1_4_1. If users plan to use BIDS format, the workflow can still be applied as DataLad is BIDS compatible. However, as BIDS is sensitive to specific characters, e.g. hyphens and underscores, the IDs should contain only numbers and letters. A possible alternative for **SP_T1_4_1** would read **SPsT1c4m1**, which replaces the underscores with the initial letters of subproject (s), cage (c), and mouse (m). Note: if special characters were included in the study IDs in the past, making the files BIDS-compatible requires much more attention, e.g. writing the correct information also in the NIfTy header and in all related metadata files.

#### File name

Whenever possible, the elements of the file names are either generated automatically or should be assigned according to a standardized scheme, which includes the StudyID, test/experiment, and the time point (if applicable). This way, the file name will be unique and contains already essential metadata. As an example, SPs1c4m1CytD4, would relate to the Cylinder Test (Cyt), a mouse behavior test, which was performed on day 4 (D4) with study ID SPs1c4m1. The file/folder paths are stored in the electronic database^13^ along with important metadata information about the experiment. In the case of MRI, this includes the anesthesia protocol (including the exact timing and dosage), configuration details (e.g. coil, gradient), and the list of MRI scans.

According to the basic rules of data storage^30^, open data formats are used whenever possible. Our datasets contain a wide range of files, ranging from small text files to larger MRI files and video recordings. There is no general restriction in the file format to be compatible with the workflow (Table 2).

**Table 2:** Typical file formats in our project.

Data types	Description	DataLad tracking
.txt	Text format	Holistic
.xlsx	Microsoft Excel spreadsheets	Difference
.csv	Delimited text file	Holistic
.docx	Microsoft Word documents	Difference
.nii	File format for neuroimaging	Holistic
.gz	compressed archive	Difference
.rar	compressed archive	Difference
.py	file containing code written in Python	Holistic
.mat	binary MATLAB® files that store workspace variables	Difference
.m	file containing code written in MATLAB	Holistic
.png	image format with lossless compression	Difference
.jpg	image format with lossy compression	Difference
.lif,.vsi,.zvi	vendor-specific microscopy file format (Leica, Olympus, Zeiss)	Difference
.yml	configuration files	Holistic
.json	open standard file format and data interchange format	Holistic

INFO: For any text file format (e.g. txt, csv, json) DataLad tracks the changes in the file on a line-by-line basis (*holistic)*. As a result, each line in e.g. Python code can be attributed to a commit (and author) that changed it last (using Git functionality). For all other data formats, DataLad tracks the *difference* on a per-file basis using file checksums, i.e., information on when and who changed the document is stored but not which part of the file changed. In terms of Open Science and long-term usability, it is recommended to use line-by-line file types (machine-readable, e.g. csv) whenever possible.

## Supplementary information

Supplementary_Kalantari_Paper

## Data Availability

We recommend using the test dataset^27^ (10.12751/g-node.3yl5qi). It contains a basic yoda structure (Fig. 2A) and is sufficiently small to allow rapid processing.

## Code availability

DataLad and GIN are freely available. The manuscript contains all code to reproduce the workflow.
