# Supplementary material for: How to establish and maintain a multimodal animal research dataset using DataLad
Source: Sci Data. 2023 Jun 5;10:357. doi: 10.1038/s41597-023-02242-8 (PMC10241774; doi:10.1038/s41597-023-02242-8)
Supplement: Supplementary file 1 — Supplementary_Kalantari_Paper [file 41597_2023_2242_MOESM1_ESM.pdf]

Supplementary Material

Video guide

DataLad-Cloning.mp4
DataLad-Creating.mp4

Software Installation

CRITICAL

OS dependencies

All operating systems were built with different purposes and therefore all of them have their special advantages and caveats compared to each other. Here we summarize installation routines for the most popular operating systems, i.e., Windows, Mac, and Linux. A note of caution: pay attention to every detail of the workflow, especially if you want to apply this work to a repository of your own. For the most up-to-date installation instructions, see the related websites. Get familiar with the command line/terminal before starting the installation process (https://ubuntu.com/tutorials/command-line-for-beginners#1-overview. Command line entries are highlighted by `>`.

WINDOWS

- Python 3.6.x (https://www.python.org/downloads/release/python-3107)

Files

Version	Operating System	Description	MD5 Sum	File Size	GPG	Sigstore	
Gzipped source tarball	Source release		1aea68575c0e97bc83ff8225977b0d46	26006589	SIG	CRT	SIG
XZ compressed source tarball	Source release		b8094f007b3a835ca3be6bdf8116cccc	19618696	SIG	CRT	SIG
macOS 64-bit universal2 installer	macOS	for macOS 10.9 and later	4c89649f6ca799ff29f1d1dffcbb9393	40865361	SIG	CRT	SIG
Windows embeddable package (32-bit)	Windows		7e4de22bfe1e6d333b2c691ec2c1fcee	7615330	SIG	CRT	SIG
Windows embeddable package (64-bit)	Windows		7f90f8642c1b19cf02bce91a5f4f9263	8591256	SIG	CRT	SIG
Windows help file	Windows		643179390f5f5d9d6b1ad66355c795bb	9355326	SIG	CRT	SIG
Windows installer (32-bit)	Windows		58755d6906f825168999c83ce82315d7	27779240	SIG	CRT	SIG
Windows installer (64-bit)	Windows	Recommended	bfbe8467c7e3504f3800b0fe94d9a3e6	28953568	SIG	CRT	SIG

https://www.python.org/downloads/release/python-3107/

I. Download the python installer according to figure 2

Figure 2: Windows installer (64-bit) or (32-bit)

II. When running the installer, make sure to select *Add Python to PATH* option, as this is required for subsequent installation steps and interactive use later on. Other than that, using the default installation settings is just fine.

III. Verify installation by opening the terminal and running the command `python`, the output should look like this: `Python 3.9.7 (default, Sep 16 2021, 16:59:28) [MSC v.1916 64 bit (AMD64)] :: Anaconda, Inc. on win32 Type "help", "copyright", "credits" or "license" for more information.`

- Git (https://git-scm.com/download/win)
  I. Download the Git installer for (64-bit) or (32-bit) windows OS.

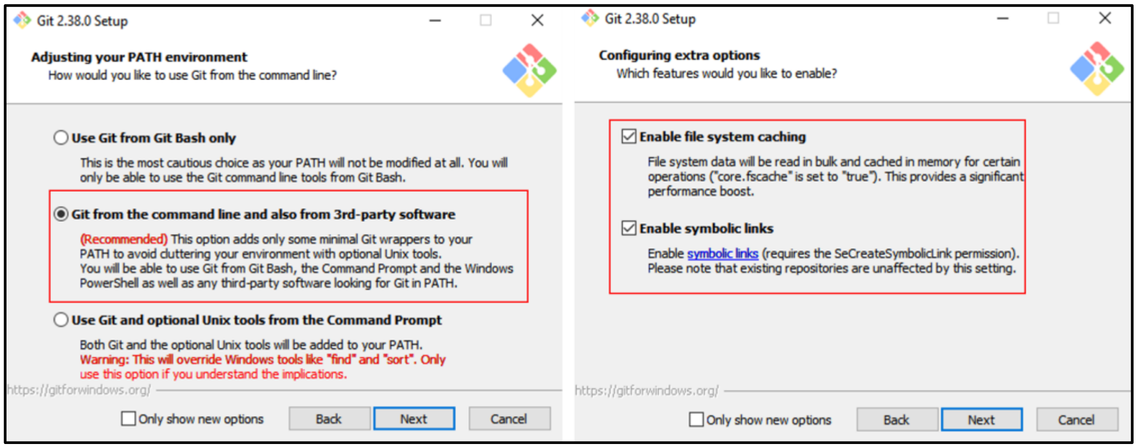

  II. When installing Git configure the specific settings shown in figure 3

Figure 3: 1) Select Git from the command line and also from 3rd-party software 2) Enable file system caching 3) Enable symbolic links

- Git-annex (Using datalad-installer)
  I. The most convenient way to deploy git-annex is via the DataLad installer. Once Python is available, it can be installed with the Python package manager pip. Open a command prompt and run:

```
> pip install datalad-installer
```

  II. Open another command prompt in administrator mode and run:

```
> datalad-installer git-annex -m datalad/packages
```

  This will download a recent git-annex, and configure it for your Git installation. The admin command prompt can be closed afterward, all other steps do not need it. For performance improvements, we recommend also setting the following git-annex configuration:

```
> git config --global filter.annex.process "git-annex filter-process"
```

- Datalad
  With Python, Git, and git-annex installed, DataLad can be installed, and later also upgraded using pip by running:

```
> pip install datalad
```

- 7-Zip (https://7-zip.org/)
  Download it from the 7-zip website (64bit installer when in doubt), and install it into the default target folder.
- nano Text editor (https://files.lhmouse.com/nano-win/)
  I. Download the latest version of the program (.7z file)
  II. Extract the OS-specific version
  III. Copy the `nano.exe` file from `~\pkg_x86_64-w64-mingw32\bin\nano.exe` to `C:\Windows\`.
  IV. Verify installation by running the command `nano` in a new terminal.
  Tip: The caret sign “^” means the control key on the keyboard.

Mac

On Mac, it is recommended to install a separate version of Python and Datalad in an Anaconda environment. This prevents conflicts with the existing Python version on Mac OS. Make sure you choose the correct installation version, especially if you are using the new Apple M1 chip.

1. Install XCode from the Apple App Store (https://apps.apple.com/us/app/xcode/id497799835)
2. Install the package manager Homebrew (https://brew.sh)
3. Download and install Anaconda (https://docs.anaconda.com/anaconda/install/mac-os/)
   In the terminal: create a new Conda environment for this workflow
   ```
   >conda create -n workflow python=3.10
   ```
   (-n specifies the name of the workflow)
   During the process, Conda will automatically determine the necessary packages to be installed. If asked to proceed, type: `y` (for yes). The installation should finalize with instructions on how to activate/deactivate the environment.
   ```
   >Preparing transaction: done
   >Verifying transaction: done
   >Executing transaction: done
   >#
   ># To activate this environment, use
   >#
   >#     $ conda activate workflow
   >#
   ># To deactivate an active environment, use
   >#
   >#     $ conda deactivate
   ```
   Activate the environment:
   ```
   >conda create -n workflow python=3.10
   ```

4. Register to GIN and install the command line tool (https://gin.g-node.org/G-Node/Info/wiki/GIN+CLI+Setup#macos)
   ```
   >brew tap g-node/pkg
   >brew install g-node/pkg/gin-cli
   ```

5. Install DataLad
   `>brew install datalad`

Linux: (Neuro) Debian, Ubuntu, and similar systems

1. Install DataLad and all dependencies (including git-annex and p7zip)
   `>sudo apt-get install datalad`
